# Patient- and 3D morphometry-based nose outcomes after skeletofacial reconstruction

**DOI:** 10.1038/s41598-020-61233-6

**Published:** 2020-03-06

**Authors:** Rafael Denadai, Pang-Yun Chou, Hyung Joon Seo, Daniel Lonic, Hsiu-Hsia Lin, Betty C. J. Pai, Lun-Jou Lo

**Affiliations:** 1 Department of Plastic and Reconstructive Surgery and Craniofacial Research Center, Chang Gung Memorial Hospital, Chang Gung University, Taoyuan, Taiwan; 2 Department of Plastic and Reconstructive Surgery, Pusan National University Hospital, Busan, Korea; 3 Centre of Plastic, Aesthetic, Hand and Reconstructive Surgery, University of Regensburg, Franz-Josef-Strauß-Allee, Regensburg, Germany; 4 Department of Plastic and Reconstructive Surgery, Helios Hospital München West, Munich, Germany; 5 Image Lab and Craniofacial Research Center, Chang Gung Memorial Hospital, Taoyuan, Taiwan; 6 Department of Craniofacial Orthodontics and Craniofacial Research Center, Chang Gung Memorial Hospital, Taoyuan, Taiwan

**Keywords:** Three-dimensional imaging, Outcomes research

## Abstract

Patient satisfaction with the shape and appearance of their nose after orthognathic surgery-based skeletofacial reconstruction is an important, but often overlooked, outcome. We assessed the nose-related outcomes through a recently developed patient-reported outcome instrument and a widely adopted 3D computer-based objective outcome instrument, to verify any correlation in the results produced by these tools. We collected FACE-Q nose appearance reports (2 scales) and 3D nasal morphometry (10 parameters) from patients with class III skeletal pattern and congenital cleft lip palate deformity (n = 23) or developmental dentofacial deformity (n = 23) after (>12 months) skeletofacial reconstruction. The cleft and dentofacial cohorts demonstrated significantly (p < 0.001) poorer satisfaction scores with regard to the FACE-Q nostrils scale than the normal age-, gender-, and ethnicity-matched subjects (n = 107), without any significant difference in FACE-Q nose scale. The cleft cohort had significantly (p < 0.001) smaller nasal length, nasal tip projection, and columellar angle and greater nasal protrusion, alar width, and columellar–labial angle values than the dentofacial and normal cohorts; however, there were no significant differences between the dentofacial versus normal cohorts. The FACE-Q nose and nostrils scales were significantly (p < 0.001; r = −0.26–0.27) correlated to the results of the 3D morphometric analysis, with regard to nasal length, alar width, columella angle, and columellar–labial angle parameters. This study revealed differences in satisfaction with the appearance of the nose according to the type of underlying deformity, and demonstrated a significant correlation (low correlation coefficients) between the patient-reports and 3D image-based outcome measure tools, which has implications for multidisciplinary-centered research, auditing, and clinical care.

## Introduction

Facial deformities associated with abnormal maxillo-mandibular relationships, such as congenital cleft lip and palate and developmental dentofacial deformities, significantly affect oral function and facial aesthetics^1,2^. Skeletofacial reconstruction using orthognathic surgery principle is a successful treatment modality for these abnormalities^3–5^. However, a number of post-surgical facial modifications are the primary causes of concern among the patients, particularly changes in the nasal morphology^6^. A growing number of studies have addressed this issue by adopting three-dimensional (3D) nasal measurements^6–15^, but the effects of skeletofacial reconstruction are beyond these imaging-guided outcome metrics^1,4,5^. The patient satisfaction with the appearance of their nose is an important, albeit often overlooked, outcome parameter after orthognathic surgery. Therefore, including a patient’s self-perception by employing patient-reported outcome (PRO) techniques may help clinicians to understand the health-related concerns of the patient^16,17^.

Literature reviews^4,5^ have revealed that a plethora of PRO instruments have been utilized to primarily verify quality of life or oral function, with a small number of studies that specifically address the nasal appearance. FACE-Q, a cross-culturally validated and condition-specific PRO tool, provides nasal appearance-specific scales^17^. However, FACE-Q nasal scales has sporadically been adopted in facial bone reconstructive studies^18–21^.

In this setting, only few studies have satisfactorily incorporated both image- and PRO-guided outcome measurements as part of same study design^6,8^. Currently, there have been no studies that adequately describe nasal-related outcome using both 3D morphometry and FACE-Q tools. Application of 3D image- and FACE-Q-based outcome metrics in a cohort of patients who underwent facial bone surgical interventions may boost perioperative care pathways grounded on multi-professional cooperation, including psychiatrists, psychologists, dentists, orthodontists, and surgeons. Appraising the postoperative status of treated patients may provide valuable information to enhance treatment strategies, guide future care, improve informed consent, and allow patient-centered adjustments to current practice.

The primary aim of this study was to assess the post-skeletofacial surgery treatment outcomes using FACE-Q nasal appearance reports and 3D nasal morphometric analysis in two cohorts of Taiwanese Chinese patients with Class III skeletal pattern who had clefts or developmental dentofacial deformity. The secondary aims were to compare these outcomes to those in normal Taiwanese Chinese individuals, and to verify the presence or absence of correlations between PRO- and image-based outcome metrics. We hypothesized that patients with clefts would present lower FACE-Q scores than those with dentofacial deformities, patients with clefts would present lower FACE-Q scores than normal individuals, and the correlations between the FACE-Q nasal scales and 3D nasal morphometric analysis would be low or non-significant.

A primary outcome is expected in this study:

The primary endpoint of the study is the comparison of FACE-Q- and 3D nasal morphometry-based outcomes between patients with clefts (cleft cohort) and dentofacial deformity (dentofacial cohort).

The null hypothesis was:

No difference of FACE-Q- and 3D nasal morphometry-based outcomes exists between cleft and dentofacial cohorts.

## Results

A total of 46 patients (22.0 ± 1.8 years of age at data collection, 52.2% women, 50% with clefts, and each had received a two-jaw surgery with maxillary advancement, mandible setback, and pitch clockwise rotation) and 107 normal, age-, gender-, and ethnicity-matched individuals were included in this study (Table 1).

**Table 1:** Characteristics of Patients and Normal Individuals.

Parameters	Cleft cohort	Dentofacial cohort	Normal cohort	p*	p**	*p*†
**Participants** *n*	23	23	107	—	—	—
**Age** (y) m ± sd	21.7 ± 2.1	22.2 ± 1.7	22.6 ± 1.0	0.879	0.530	0.792
**Females** *n* (%)	12 (52.2)	12 (52.2)	54 (50.5)	—	0.843	0.843
**Skeletal pattern** *n* (%)						
Class I	0 (0)	0 (0)	107 (100)	—	—	—
Class III	23 (100)	23 (100)	0 (0)	—	—	—
**Two-jaw surgery** *n* (%)	23 (100)	23 (100)	—	—	—	—

*n*, number of individuals; *y*, years; m, mean; sd, standard deviation; *p*, p-value; —, not applicable; *, cleft cohort versus dentofacial cohort comparisons; **, cleft cohort versus normal cohort comparisons; †, dentofacial cohort versus normal cohort comparisons.

### FACE–Q instrument

**Figure 1:**
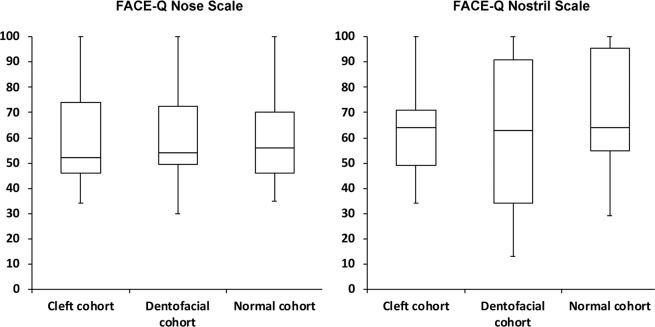
Box plots demonstrating the distribution of FACE-Q scores in the cleft, dentofacial, and normal cohorts.

The cleft and dentofacial cohorts had significantly (p < 0.001) lower scores for satisfaction in the nostrils scale than the normal cohort. The dentofacial cohort demonstrated a significantly (p < 0.001) lower score in the nostrils scale than the cleft cohort. We did not observe any significant differences when comparing satisfaction for the nose scale (Table 2; Fig. 1).

**Table 2:** FACE-Q Nasal Reports.

Parameters	Cleft cohort	Dentofacial cohort	Normal cohort	p*	p**	*p*†
**FACE-Q scales** m ± sd (95% CI)						
Nose	61.3 ± 23.4 (51.2–71.4)	62.4 ± 21.7 (53–71.8)	60.8 ± 19.3 (55.5–66.0)	0.620	0.579	0.314
Nostrils	64.2 ± 22.6 (54.4–74.0)	60.1 ± 29.5 (47.3–72.8)	69.5 ± 22.8 (63.3–75.6)	**<0.001**	**<0.001**	**<0.001**

m, mean; sd, standard deviation; mm, millimeters; °, degrees; *p*, p-value; CI, confidence interval; *, cleft cohort versus dentofacial cohort comparisons; **, cleft cohort versus normal cohort comparisons; †, dentofacial cohort versus normal cohort comparisons. Bold values indicate statistical significance after Bonferroni correction.

### 3D nasal morphometric analysis

**Figure 2:**
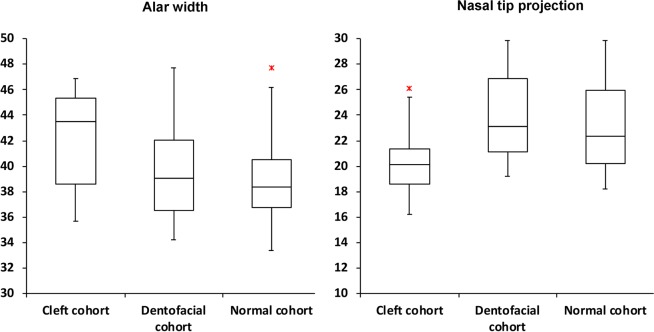
Box plots demonstrating the distribution of 3D alar width and nasal tip projection values in the cleft, dentofacial, and normal cohorts. Red asterisks indicate maximum outliers’ values.

**Figure 3:**
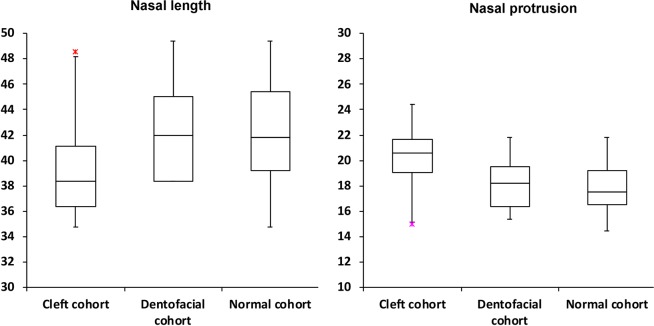
Box plots demonstrating the distribution of 3D nasal length and nasal protrusion values in the cleft, dentofacial, and normal cohorts. Red asterisks indicate maximum outliers’ values.

**Figure 4:**
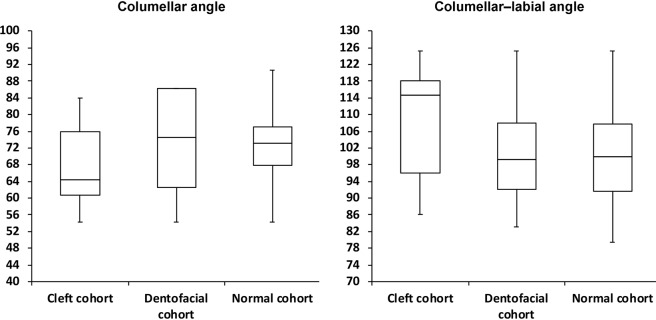
Box plots demonstrating the distribution of 3D columellar angle and columellar-labial angle values in the cleft, dentofacial, and normal cohorts.

The cleft cohort had significantly (p < 0.001) smaller nasal length, nasal tip projection, and columellar angle parameters and greater nasal protrusion, alar width, and columellar–labial angle parameters values than the dentofacial and normal cohorts. There were no significant differences in the nasal height, nasal dorsum angle, nasal surface area, and nasal volume parameters. Dentofacial and normal cohorts revealed no significant difference for all tested parameters (Table 3; Figs. 2–4; Supplementary Figs. S1–S4).

**Table 3:** 3D Nasal Morphometric Analysis.

Parameters	Cleft cohort	Dentofacial cohort	Normal cohort	p*	p**	*p*†
**3D morphometry** m ± sd (95% CI)						
Nasal length (mm)	39.6 ± 4.0 (37.8–41.3)	42.7 ± 3.8 (41.1–44.3)	42.2 ± 3.7 (41.2–43.2)	**<0.001**	**<0.001**	0.632
Nasal height (mm)	49.3 ± 3.1 (47.9–50.6)	49.9 ± 4.0 (48.2–51.6)	49.6 ± 3.8 (48.5–50.7)	0.528	0.650	0.774
Nasal protrusion (mm)	20.2 ± 2.4 (19.2–21.3)	18.1 ± 1.8 (17.3–18.9)	17.8 ± 1.6 (17.3–18.2)	**<0.001**	**<0.001**	0.761
Alar width (mm)	42.2 ± 3.8 (40.5–43.8)	39.6 ± 4.1 (37.8–41.3)	38.8 ± 3.0 (38.0–39.7)	**<0.001**	**<0.001**	0.407
Nasal tip projection (°)	20.1 ± 2.6 (18.9–21.2)	23.8 ± 3.4 (22.4–25.3)	23.0 ± 3.2 (22.1–23.9)	**<0.001**	**<0.001**	0.624
Nasal dorsum angle (°)	21.1 ± 2.4 (20.1–22.2)	20.7 ± 2.5 (19.7–21.8)	20.3 ± 2.1 (19.7–20.9)	0.479	0.693	0.587
Columellar angle (°)	67.1 ± 10.0 (62.8–71.4)	73.5 ± 11.6 (68.5–78.5)	72.5 ± 7.5 (70.6–76.9)	**<0.001**	**<0.001**	0.456
Columellar–labial angle (°)	107.6 ± 12.7 (102.1–113.1)	99.7 ± 10.4 (95.2–104.2)	100.0 ± 10.3 (97.1–102.9)	**<0.001**	**<0.001**	0.793
Nasal surface area (cm^2^)	25.2 ± 5.1 (23.0–27.4)	26.4 ± 4.6 (24.4–28.4)	25.9 ± 4.7 (24.6–27.2)	0.323	0.516	0.662
Nasal volume (cm^3^)	8.0 ± 2.4 (7.0–9.1)	8.3 ± 2.5 (7.2–9.3)	8.1 ± 2.3 (7.4–8.7)	0.771	0.928	0.854

m, mean; sd, standard deviation; mm, millimeters; cm, centimeter; °, degrees; *p*, p-value; CI, confidence interval; *, cleft cohort versus dentofacial cohort comparisons; **, cleft cohort versus normal cohort comparisons; †, dentofacial cohort versus normal cohort comparisons. Bold values indicate statistical significance after Bonferroni correction.

### Correlations

We noted significant (p < 0.001; r = −0.26–0.27) correlations between the FACE-Q satisfaction for the nose and nostrils scales, along with the 3D nasal length, alar width, columella angle, and columellar-lip angle parameters in both cleft and dentofacial cohorts. No significant correlation was observed for the remaining tested parameters (Table 4).

**Table 4:** Correlations for Cleft and Dentofacial Cohorts.

3D morphometry	FACE − Q scales			
	Cleft cohort		Dentofacial cohort	
	Nose	Nostrils	Nose	Nostrils
	*r* (*p*)	*r* (*p*)	*r* (*p*)	*r* (*p*)
Nasal length	0.18 (**<0.001**)	0.27 (**<0.001**)	0.16 (**<0.001**)	0.25 (**<0.001**)
Nasal height	0.39 (0.941)	0.21 (0.806)	0.02 (0.473)	0.42 (0.637)
Nasal protrusion	0.26 (0.540)	0.03 (0.739)	0.28 (0.307)	0.06 (0.910)
Alar width	−0.10 (**<0.001**)	−0.26 (**<0.001**)	−0.11 (**<0.001**)	−0.19 (**<0.001**)
Nasal tip projection	0.38 (0.482)	0.08 (0.205)	0.37 (0.623)	0.06 (0.413)
Nasal dorsum angle	0.23 (0.603)	0.29 (0.185)	0.20 (0.780)	0.34 (0.275)
Columellar angle	0.21 (**<0.001**)	0.14 (**<0.001**)	0.09 (**<0.001**)	0.12 (**<0.001**)
Columellar–labial angle	−0.25 (**<0.001**)	−0.19 (**<0.001**)	−0.15 (**<0.001**)	−0.23 (**<0.001**)
Nasal surface area	0.04 (0.946)	0.33 (0.715)	0.07 (0.582)	0.20 (0.307)
Nasal volume	0.09 (0.841)	0.15 (0.903)	0.31 (0.266)	0.04 (0.838)

*r*, correlation coefficient; *p*, p–value. Bold value indicates statistical significance after Bonferroni correction.

The linear regression analyses revealed significant correlations (p < 0.001) between gender and seven nasal morphometric parameters (nasal length, nasal height, alar width, nasal protrusion, nasal tip projection, nasal surface area, and nasal volume), with no significant correlation between gender and FACE-Q scales. Significant correlations (p < 0.001) were observed between group and six nasal morphometric parameters (nasal length, alar width, nasal protrusion, nasal tip projection, columellar angle, and columellar–labial angle) and between group and a FACE-Q parameter (nostrils scale). No significant correlation was observed between age and nasal morphometric and FACE-Q scales (Table 5).

**Table 5:** Linear Regression Analyses.

Dependent variables	Independent variables										
	Age				Gender (Male versus Female)				Groups (Cleft versus Dentofacial cohorts)		
	*β* (95% CI)	*S*	*p*	*β* (95% CI)		*S*	*p*	*β* (95% CI)		*S*	*p*
**FACE-Q scales**											
Nose	0.23 (−0.46–0.92)	0.81	0.649	1.07 (−0.36–2.5)		0.34	0.526	0.38 (−1.57–2.33)		0.16	0.720
Nostrils	0.50 (−0.76–1.76)	0.64	0.481	3.90 (2.94–4.86)		0.51	0.805	−0.52 (−0.79 – −0.25)		0.37	<0.001
**3D nasal morphometry**											
Nasal length	3.71 (1.53–5.89)	1.54	0.570	2.63 (1.52–3.74)		0.67	<0.001	0.99 (0.28–1.72)		0.83	<0.001
Nasal height	1.38 (−0.29–3.05)	1.02	0.336	1.85 (1.18–2.52)		1.31	<0.001	0.41 (−0.61–1.43)		0.74	0.639
Nasal protrusion	1.93 (0.84–3.02)	1.07	0.823	1.42 (0.49–2.35)		0.50	<0.001	−0.84 (−1.53 – −0.15)		0.30	<0.001
Alar width	0.18 (−0.76–1.12)	0.83	0.397	3.50 (1.88–5.12)		1.68	<0.001	−0.73 (−1.14 – −0.32)		0.62	<0.001
Nasal tip projection	2.63 (0.53–4.73)	1.56	0.732	1.97 (0.76–3.18)		0.82	<0.001	0.56 (0.15–0.97)		0.49	<0.001
Nasal dorsum angle	0.50 (−0.39–1.39)	0.98	0.627	2.79 (0.90–4.68)		1.63	0.710	−0.17 (−0.91–0.57)		0.38	0.403
Columellar angle	3.94 (2.77–5.11)	2.10	0.861	0.94 (−0.48–2.36)		1.39	0.503	0.30 (0.19–0.41)		0.15	<0.001
Columellar–labial angle	1.29 (−0.11–2.69)	1.66	0.608	2.43 (−0.20–5.06)		0.25	0.948	−0.65 (−1.19 – −0.11)		0.26	<0.001
Nasal surface area	1.80 (−0.16–3.76)	1.74	0.745	2.29 (1.51–3.07)		1.06	<0.001	0.49 (−0.41–1.39)		0.67	0.925
Nasal volume	0.47 (−0.54–1.48)	0.89	0.916	4.90 (2.44–7.36)		1.73	<0.001	0.22 (−0.39–0.83)		0.48	0.681

β, regression beta coefficient; CI, confidence interval; S, standard error of the regression; *p*, p–value. Encoding: female = 0 and male = 1; cleft cohort = 0 and dentofacial cohort = 1.

## Discussion

Selecting an appropriate tool to design a valid and meaningful study is imperative, as it directly influences the value of outcome-based research^22–27^. Studies examining the nose-related outcomes associated with skeletofacial reconstruction have primarily adopted the 3D morphometric-based objective measure tool^6^. Although these objective outcomes are important, nose appearance is subjective, and incorporating patients’ opinion about the same is of paramount importance^17,28^.

A range of instruments has been adopted in PRO-based research^4,5,7,8^. However, the lack of reliability or validity has impaired the interpretation or further application of a number of these adopted tools^4,5^. Additionally, most of these studies have employed generic instruments that were not designed to isolate the necessary areas of concern to specific patient populations^4,5^. The Modified Orthognathic Quality of Life Questionnaire presents the nose/lip aesthetics domain, but the four items combine the nose and lip issues; therefore, lower nose-related values may be leveled out by higher lip-related ones to create the mean value for the domain^8^.

In this study, we applied two outcome tools (FACE-Q^17^ and 3D nasal morphometry^29–33^) to identify nose-related outcomes from two cohorts of treated patients who were matched for age, gender, ethnic, and the type of skeletal relationships, but presented distinctive underlying abnormalities (clefts and dentofacial deformities). We enrolled matched healthy individuals that enabled us to make valid deductions from the tested comparisons. To further comprehend the clinical and scientific performance of FACE-Q, we tested if it could be correlated with a widely adopted 3D morphometry tool.

Overall, our 3D findings were similar to previous studies comparing cleft and non-cleft populations^15,29,34,35^. Patients with clefts demonstrated three significant dissimilarities to the other cohorts from a clinical standpoint, which included the cephalic rotation of the nasal tip (numerically represented by smaller columella angle and nasal length and greater columellar–labial angle and nasal protrusion), insufficient nasal tip projection (smaller nasal tip projection), and greater alar width. It reinforced that mature patients with clefts show morphological differences in the nasal soft tissue than normal individuals, regardless of surgical management of the skeletal framework^29^.

Previous studies revealed that modifications to the alar width have been the nasal parameter that was more consistently reported after orthognathic surgery, with columella- and nostrils-related measurements also being frequently investigated^6^. Interestingly, most of the significant correlations were associated with these particular aspects. FACE-Q nose and nostrils scales showed significant correlations to the 3D alar width, columella angle, and columellar–labial angle parameters for both patient cohorts, which clinically represent wider nasal base, short nose, and tip up-rotation with the nostrils shown on frontal view^15,29,36–38^. However, it only presented low correlation coefficients, suggesting that the tested measure tools are only marginally connected and other potential explanatory factors were involved in this outcome measurement process.

FACE−Q and 3D image tools differentiated the enrolled cohorts for most of tested variables, while being consistent with previous findings^15,29,34,35^. Therefore, it may be possible that the presence of poor or the lack of significance for the tested correlations may not be associated to the incapacity of each tool to detect relevant features from the patient (nose and nostril appearance) and the abnormalities (clefts versus dentofacial deformities versus normal individuals). Studies have reported that the appraisal of correlations between two different outcome measurement tools has implications for the ongoing discussions regarding the correct interpretation and application of each existing tool in clinical and research settings^18,39,40^. Therefore, further investigation is important to improve our understanding of the assessed tools by testing further predefined hypotheses about expected correlations, as it would attenuate the risk of bias and assist researches to avoid substitute justifications after data analysis, as defined before our data collection.

The alar width- and nostrils-related features are key factors when the characteristics of Asian noses are placed into ethnical and cultural perspectives^15,29,34,35^. Unlike anatomical norms for Caucasian noses, Asians exhibit a flatter dorsum, a wider alar width, and a short nose in which there is much upward tilt of the nasal tip along with an increased nostril exposure^15,29,34,35^. Accordingly, Asians frequently request surgery to correct their typical, but not fully acceptable nose^36–38^. Moreover, surgical maneuvers have been performed to attenuate these specific nasal changes following skeletofacial reconstruction, including the overcorrected alar cinch suture^41^. It may justify, at least partly, the absence of significant 3D morphometric differences between the dentofacial and normal cohorts. As the patients with clefts have a characteristically increased alar width than those with dentofacial deformity preoperatively^29^, the overcorrected cinch suture did not have the same effect in maintaining the parameters of the alar width similar to normal individuals.

These cultural and ethnic elements may have influenced the answers provided by patients and normal individuals to a FACE-Q questionnaire. Therefore, the nasal morphological features and the effect of surgical intervention were the predominantly differentiating factors among the types of underlying deformities. These factors may partially elucidate the lower FACE-Q scores presented by the cleft cohort than normal individuals, which included the greater 3D morphologic differences (deformities) and poorer FACE-Q scores. However, this do not explain the reason behind the relatively high scores achieved by the cleft cohort than the dentofacial cohort for the FACE-Q nostrils scale, despite the presence of significant differences in several 3D parameters. Contrary to our initial hypothesis, which predicted that the cleft cohort would have poorer FACE-Q scores than their peers due to their nose-related concerns being inherently connected with their underlying deformity since infancy^2,31^. Previous studies revealed mixed results, demonstrating that patients with clefts stated that their noses were either better or worse after skeletofacial surgical management than those with dentofacial deformities^8,15^. We hypothesized that as patients with clefts have received longitudinal multidisciplinary support since their infancy, they would have incorporated coping skills that helped them judge the surgical-induced nasal changes along with the presence of nasal deformities in a relatively positive perspective than their peers with dentofacial deformities^2,31,42–44^.

This study was not without limitations. Since no prior investigation employed the FACE-Q questionnaire and 3D morphometry for analysis of a cohort of patients, we could not perform a direct comparative appraisal of the current versus former findings. The generalizability of our findings cannot be assumed, as all included patients were treated by particular procedural strategies, including the digital occlusion set up, virtual surgery, surgery–first model, and two-jaw orthognathic surgery with single-splint technique^3,45–49^. We enrolled a restricted final sample as only matched patients and controls were included for analysis. An a priori sample size calculation could not be defined due to the methodological heterogeneity between the current and previous study designs. We also did not calculate post-hoc power analysis due to the inadequacy of this specific statistical technique.

We included a sample composed by young adult patients, with a low age range (18 to 24 years). The inclusion of patients who reached skeletal maturity and an age-matched healthy cohort has attenuated, at least partially, the bias of nasal growth-related change or aging process-associated factors when interpreting our results. The regression analysis revealed no significant correlation between age and nose-related parameters. Moreover, the regression analysis demonstrated significant correlations between gender and 3D nasal morphometric parameters, with positive regression beta coefficients (encoded with 0 = female and 1 = male) for nasal length, nasal height, alar width, nasal protrusion, nasal tip projection, nasal surface area, and nasal volume. Previous investigations have also revealed that males had larger values than females for similar 3D nasal morphometric parameters^29,50^. We also observed no significant correlation between gender and FACE-Q scales, and former studies have suggested a little bias related to gender when appraising the FACE-Q-derived data^19,20,51,52^.

As this is a cross-sectional study, the significant correlations should not be interpreted as causal relationships^53^. The present study may act as a data reference to generate hypotheses that justify supplementary scientific explorations, including investigations with multivariate analysis addressing the potential predictors (e.g., sociodemographic, cephalometric, nostril size and symmetry, and nose functional parameters) of nose-related outcomes in patients with unilateral cleft and dentofacial deformities. Auxiliary cohort compositions may also be tested, incorporating other management protocols, underlying deformities (e.g., craniofacial microsomia and skeletal Class II pattern), and nose-centered stratifications for the presence of apparent nostril show and requirement for surgical intervention to correct short nose, alar widening, or nostril show as perceived by observers, clinicians, and the patients themselves.

Our reproducibility analysis demonstrated that the 3D nasal measurements were reliable (for all measurements) and highly precise (with an overall mean absolute difference across all measurements being less than 0.5). We used a published standardized reference frame for head orientation^30^, but we did not assess reproducibility of 3D facial model orientation. Additionally, we do not provide minimally important clinical differences, deserving further investigation.

Despite these limitations, our study provides suggestions regarding the applicability of tested tools in clinical and research settings. As virtually all existing measure tools are accompanied by inherent bias and limitations^54–56^, each study design should be constructed after having a thorough judgement of restrictions and qualities of each tool using well-defined hypotheses regarding realistic endpoints of outcome-based research. We predict that FACE-Q and 3D morphometry tools would be embraced either as isolated (since the limitations are suitable for the study purpose) or combined instruments, but not as exchangeable instruments. Incorporating the FACE-Q instrument can provide additional evidence beyond that delivered by 3D computer-assisted data (and vice–versa).

Regarding clinical practice purposes, our data highlighted that it is essential for patients with clefts and dentofacial deformities to have realistic expectations about nose outcome following skeletofacial reconstruction. Multidisciplinary teams may apply our and previously published data to better prepare and educate their patients prior to surgical treatment. Patients should be counselled about the potential changes in nasal morphology after surgery, the presence of nose appearance-related concerns, and the possibility of requiring rhinoplasty during a follow-up as judged in a case-by-case manner. Stakeholders might employ our results to guide decision-making processes and investment decisions for the enhancement of therapeutic protocols of patients with skeletal abnormalities who may, for example, require psychosocial support and nose surgery to effectively deal with their nose-related issues after correcting their skeletofacial disharmony.

In conclusion, this study demonstrates that: (1) there are differences in satisfaction with the nasal appearance according to the type of underlying deformity; and (2) there is a significant correlation (low correlation coefficients) between the patient-reports and 3D image-based outcome measure tools.

## Patients and Methods

### Study population

**Figure 5:**
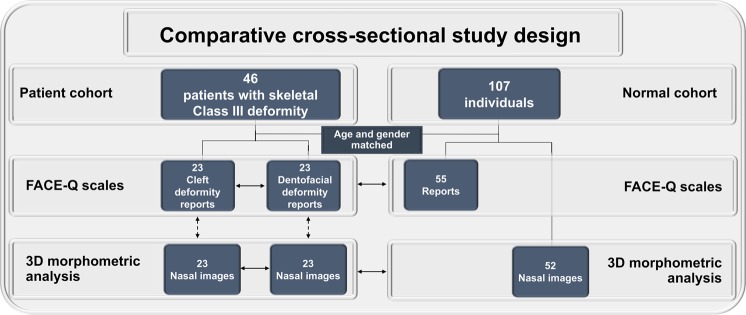
A flowchart for nose-related data collection using FACE-Q scales and 3D morphometry tools from matched patients (post-orthognathic surgery-based skeletofacial reconstruction) and normal individuals. Solid line arrows and dotted line arrows indicate comparison and correlation analyses, respectively.

This cross-sectional comparative study (Fig. 5) was performed on a cohort of Taiwanese Chinese patients aged between 18 and 24 years with Class III skeletal patterns (i.e. preoperative concave facial profile, protruding mandible, negative A point–nasion–B point angle [−4.52° ± −3.09°], and negative overjet [−5.8 mm ± −3.5 mm]) and were orthodontically and surgically treated by the two senior authors (B.C.J.P. and L.-J.L.) at the Chang Gung Craniofacial Center between 2015 and 2017. All included patients had reached skeletal maturity^57,58^ before surgical treatment.

Patient demographic (age and gender), clinical (type of underlying abnormality, such as unilateral complete cleft lip and palate deformity or developmental dentofacial deformity), surgical (type of procedure), and outcome (FACE-Q- and 3D morphometric-based nose measurements) data were collected from the Chang Gung Craniofacial Research Center database after obtaining the approval of the Institutional Review Board (Chang Gung Medical Foundation, protocol 104-A253B). All experiments and the study methods were carried out in accordance with the approved guidelines of Institutional Review Board. Informed consent from guardians was obtained for those patients who are below 20 years of age. All patients of 20 years of age or older provided their own informed consent for participation. Informed consent for publication of identifying information/images in an online open-access publication was obtained from the patient displayed in this article.

Exclusion criteria were non–native Mandarin Chinese speaker; any form of mental disabilities that would prevent them from completing the questionnaires; Class I or II skeletal patterns; any syndromic diagnosis; previous orthognathic surgery; any facial or nasal surgical intervention from the time of the procedure to data collection; and an incomplete postoperative follow-up (<12 months). Selected patients were classified into two groups based on their underlying deformity, namely cleft cohort and dentofacial cohort.

Subjects with normal Class I occlusion, proper incisor overbite and overjet (0–2 mm), and balanced facial profile were identified and included in a normal cohort, adjusted for matching factors. For this, Taiwanese Chinese individuals aged between 18 and 24 years were randomly recruited based on incidental contacts from members of the general community. Each potential participant was clinically screened extra- and intra-orally by members of multidisciplinary craniofacial team (orthodontist and plastic surgeon). To obtain an age-matched cohort, all normal, healthy individuals were selected according to specific age levels (18 to 24 years old) until at least 6 participants per age level were included for male and female groups. Exclusion criteria were non–native Mandarin Chinese speaker; any form of mental disabilities that would prevent them from completing the questionnaires; Class II or III malocclusion; presence of congenital or acquired dento-skeletofacial deformity; previous facial or nasal trauma; any history of facial, nasal, or orthodontic therapeutic intervention.

### Skeletofacial reconstructive approach

**Figure 6:**
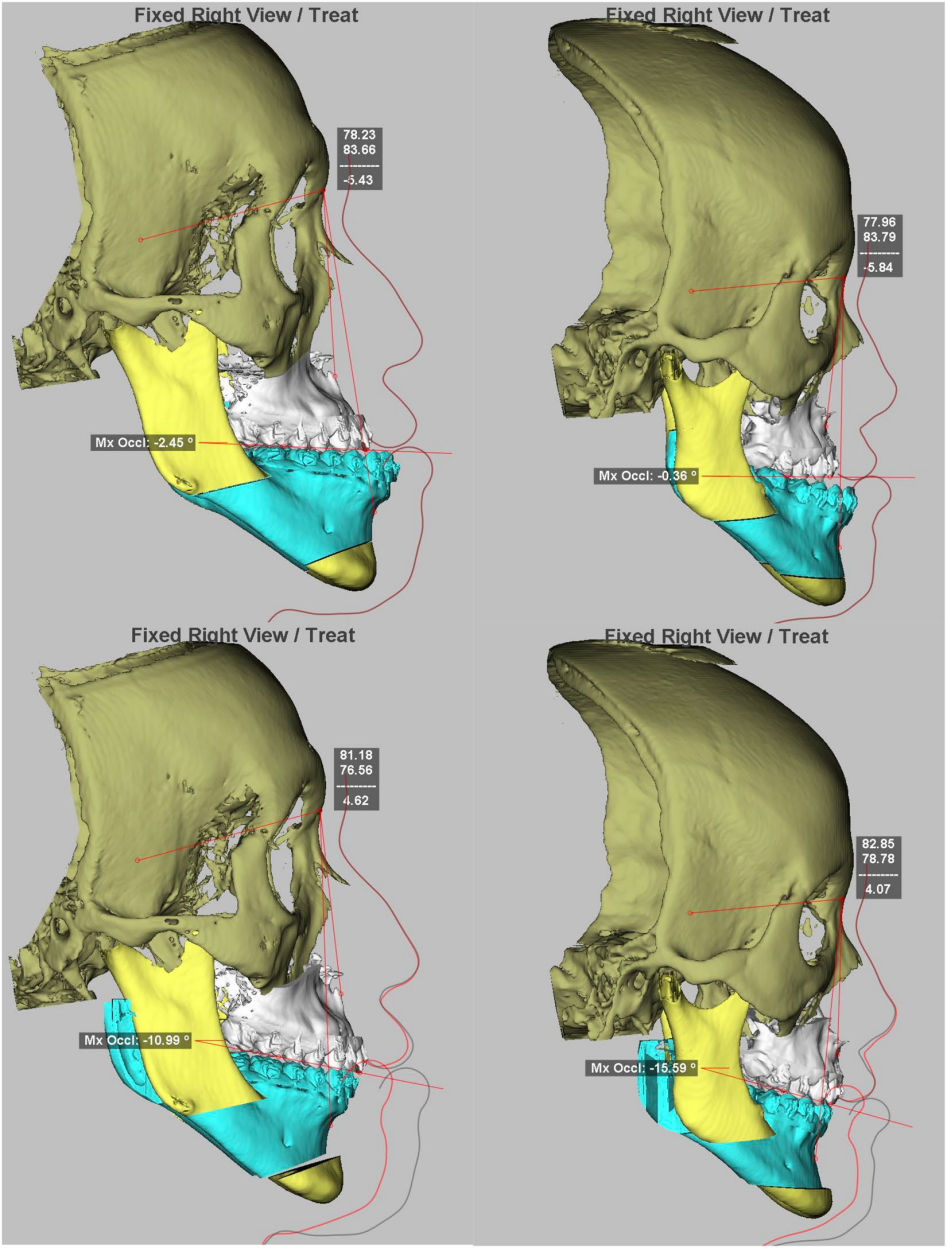
3D simulation of single-splint two-jaw skeletofacial reconstruction procedures using virtual models of a patient with congenital cleft deformity (*left*) and another patient with developmental dentofacial deformity (*right*). All included patients received a similar composition of movements (maxillary advancement, mandible setback, and pitch clockwise rotation) of the single-unit maxillomandibular complex (combined horizontal Le Fort I and mandibular bilateral sagittal splits osteotomies) as revealed by the actual Class III skeletal deformity (*top*) and the surgical planning in frontal (*center*) and profile views (*bottom*).

**Figure 7:**
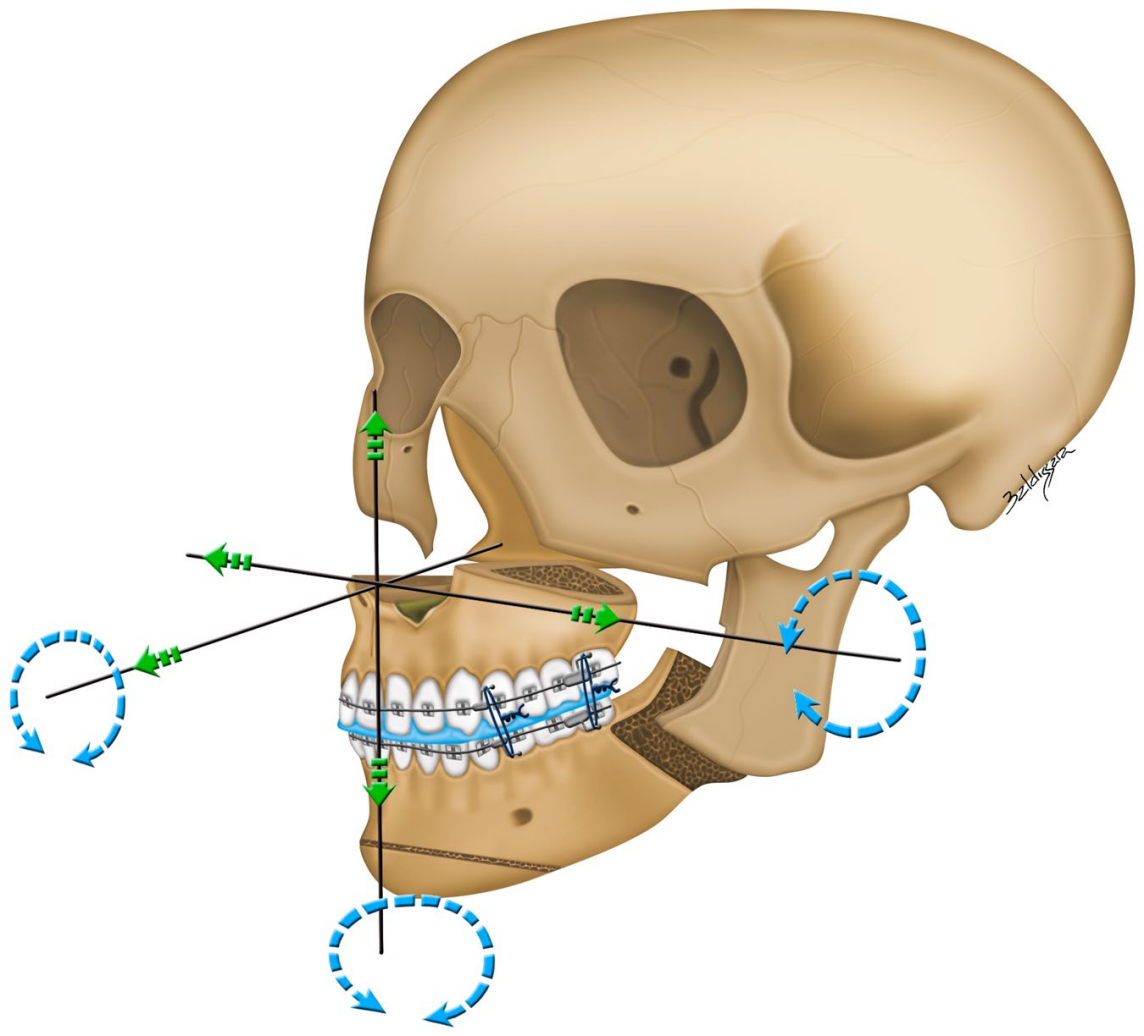
Single-splint two-jaw skeletofacial reconstruction approach. Under general anesthesia and nasotracheal intubation, the maxilla (Le Fort I segment) and mandible (two proximal ramus segments and one distal segment) were osteotomized, fixed in the final occlusal split (surgical splint in blue color), and mobilized as an integrated maxillomandibular complex. To incorporate the preoperative virtual planning (Fig. 2) in actual surgery, the 34axilla-mandibular complex was moved in six potential directions, including pitch, roll, and yaw rotations (round arrow), along with an en-bloc linear horizontal (left or right shifts and advancements or setbacks in the antero-posterior direction) and vertical (extrusion or intrusion) movements (straight arrows). Maxillary advancement, mandibular setback, and pitch clockwise rotational movements were typically used to correct the Class III skeletal deformity, with additional movements being individualized for dental occlusion and facial status of each patient. After confirmation of the midline coordination using a modified facebow device, upper incisor show, inter-commissural plane, contour symmetry, and lower face proportions, the plates and screws were rigidly affixed for medial and lateral maxillary buttresses and transcutaneous bicortical screws for proximal and distal segments of mandibular ramus. Schematic drawing prepared by Baldissara who provided a written permission to publish it under a CC BY open access license.

All patients underwent surgical reconstruction using 3D computer-assisted single-splint two-jaw technique (Figs. 6 and 7). They underwent transcutaneous alar base cinch suture with overcorrection using a non-absorbable 3–0 nylon material^41^. No trimming of the anterior nasal spine, pyriform ring, or nasal septum was performed on included patients. No postoperative intermaxillary fixation was adopted and the orthodontic therapy was initiated 2 to 4 weeks post-operatively. Full descriptions of the standard pre- and post-surgical treatment principles and details used in this center has previously been published^3,45–48^.

### FACE-Q instrument

FACE-Q^17,19,22,51,52^ is the only validated patient-derived instrument encompassing scales that measure nose appearance, which presents reliability, construct validity, discriminant validity, and responsiveness to change. The advantage of this instrument is the use of a modern psychometric method during the quantitative and qualitative developmental processes that constructed scales with specific items to map out a clinical hierarchy for the constructs of interest^17,22^. The full mixed-methods approach employed in the development and validation processes have been described in detail previously^17,19,22,51,52^. Briefly, a systematic review, interviews with patients, and input from 26 experts in the field were used to develop a conceptual framework and specific FACE-Q scales and checklists, which were further refined through cognitive interviews.

All included patients completed the Mandarin Chinese version of FACE-Q^19^ during regular clinical appointments after (>12 months) the surgical intervention. Nose (10 items) and nostril (5 items) satisfaction measurement scales were applied; these scales calculate patient satisfaction with the overall appearance of their nose and nostrils, respectively. All patients completed the measurement scales unaided and independently; they answered all questions based on their own understanding of the instructions and items. Four response options were provided, i.e. very dissatisfied, somewhat dissatisfied, somewhat satisfied, or very satisfied (1 to 4 points, respectively), for each item. Using Excel for Mac (Microsoft Corporation, Redmond, WA, USA), the total score for each scale was calculated by adding the scores of each item of that specific scale. The sum score for each scale was then converted to an equivalent Rasch transformed score, ranging from 0 to 100. Higher scores represented greater patient satisfaction with the outcome of the procedure^17,19^. This process aimed to adhere to the recommendations from the original developers of the FACE-Q^17^.

### 3D nasal morphometric analysis

All 3D imaging data were acquired using the 3dMD system (3dMD LLC, Atlanta, GA, USA) under standard conditions, including natural head position, relaxed facial musculature, and habitual dental occlusion^29,33^. The system was calibrated before every capture. Data sets were analyzed using 3dMD Vultus software package (version 2.2, 3dMD Inc., Atlanta, GA, USA).

**Figure 8:**
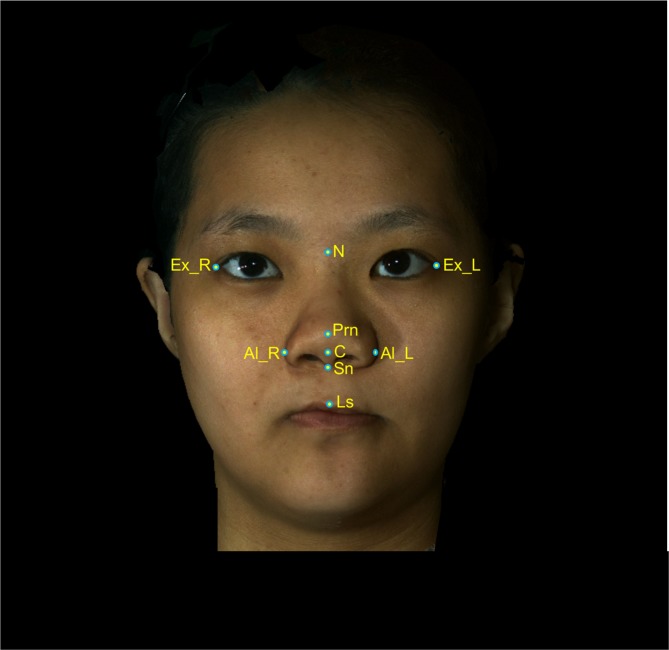
3D photogrammetric imaging of a patient with Class III skeletal pattern and cleft lip and palate deformity after single-splint two-jaw skeletofacial reconstruction showing the unilateral and bilateral anatomical landmarks: N- nasion; Prn- pronasale; C- columellar constructed point; Sn- subnasale; Al- alare; Ex- Exocanthion; Ls- Labial superius; R- right; and L- left.

**Figure 9:**
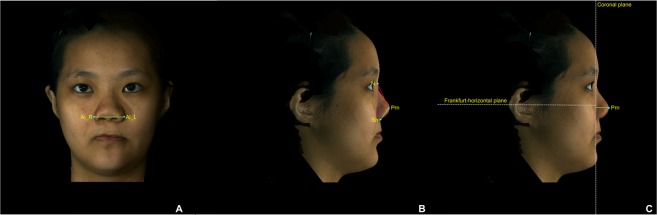
3D photogrammetric imaging displaying the nasal linear measurements, including (**A**) alar width (right Al – left Al, green line), (**B**) nasal length (N – Prn, red line), nasal height (N – Sn, purple line), nasal protrusion (Sn – Prn, green line), and (**C**) nasal tip projection (coronal plane – Prn) parameters.

**Figure 10:**
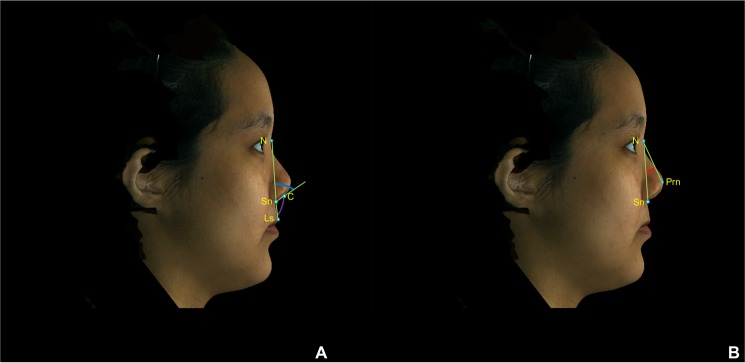
3D photogrammetric imaging displaying the nasal angular measurements, including (**A**) columellar angle (N – Sn – C, blue angle), columellar-labial angle (C – Sn – Ls, purple angle), (**B**) and nasal dorsum angle (Sn – N – Prn, red angle) parameters.

**Figure 11:**
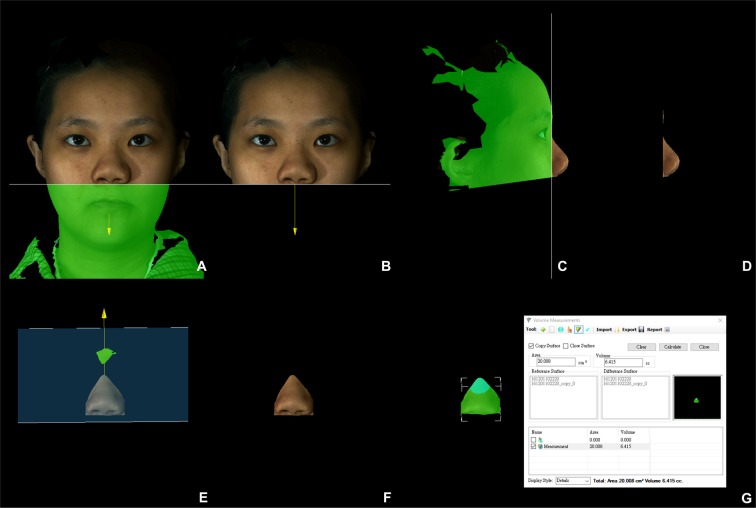
(**A–G**) 3D photogrammetric imaging displaying the nasal surface area and volume measurements, with the nose being defined as a central 3D object and regions without interest trimmed from the columellar–labial junction to the inferior border of the glabella, and from the ala, junction of the cheek, and nasal sidewall.

All anatomical landmarks, reference planes, and measurements (4 linear, 4 angular, 1 surface area, and 1 volume parameter; Table 6; Figs. 8–11) were standardized based on previous nasal morphometric studies^29–32^. We set up a uniform reference frame (horizontal, coronal, and sagittal plane) before all landmark identifications. The zoom and rotation tools were utilized to accurately identify and set the landmarks on the 3D nasal surface.

**Table 6:** Anatomical Soft Tissue Landmarks, Reference Planes, and Measurement Parameters Adopted for 3D Nasal Morphometric Analysis.

Parameters (abbreviations)	Definitions
**Landmarks**	
Nasion (N)	Most depressed midline point superior to the nasal bridge
Pronasale (Prn)	Most anterior midpoint of the nasal tip
Subnasale (Sn)	Midpoint on the nasolabial contour between the columella crest and the upper lip
Columellar constructed point (C)	Breakpoint at the end of the tangential line drawn from the Sn along the lower part of columella
Alare (Al)	Most lateral point on each alar contour
Exocanthion (Ex)	Point located at the outer commissure of each eye fissure
Labial superius (Ls)	Midpoint of the vermilion line of the upper lip
Tragion (T)	Point located at the upper margin of each tragus
**Reference planes**	
T-Ex plane	Line passing through the T and Ex points
Frankfurt-horizontal (Fh) plane	Line passing through the T point and 17.6 degrees below the Ex-T plane
**Linear measurements**	
Nasal length	Linear distance between N and Prn points
Nasal height	Linear distance between N and Sn points
Nasal protrusion	Linear distance between Sn and Prn points
Alar width	Linear distance between right Al and left Al points
Nasal tip projection	Linear distance from coronal plane intersecting the alar facial groove and perpendicular to the Fh plane to Prn point
**Angular measurements**	
Nasal dorsum angle	Angulation calculated from intersecting lines drawn from the N to Sn points and from N to Prn points (Sn – N – Prn)
Columellar angle	Angulation calculated from intersecting lines drawn from the N to Sn points and from Sn to C points (N – Sn – C)
Columellar–labial angle	Angulation calculated from intersecting lines drawn from Sn to C points and from Sn to Ls points (C – Sn – Ls)

### Reliability and precision

All 3D nasal morphometric measurements were collected in duplicate by two independent raters, with a 1-month interval between each measurement, and their mean was used for analysis.

Reproducibility (the consistency of values for repeated 3D nasal morphometric measurements) was assessed by reliability and precision. Intra- and inter-rater reliabilities (the degree of similarity between repeated measurements using the same morphometric method) were analyzed with intraclass correlation coefficient (ICC) test^59^ and were considered excellent (ICC = 0.85–0.94; Table 7) for all nasal measurements.

**Table 7:** Intra- and Inter-Rater Reliabilities for 3D Nasal Morphometric Measurements.

Parameters	Intra-rater reliability ICC (95% CI)	Inter-rater reliability ICC (95% CI)
Nasal length	0.84 (0.69–0.92)	0.89 (0.67–0.97)
Nasal height	0.87 (0.73–0.95)	0.90 (0.76–0.96)
Nasal protrusion	0.85 (0.65–0.91)	0.87 (0.73–0.93)
Alar width	0.90 (0.84–0.96)	0.94 (0.92–0.99)
Nasal tip projection	0.91 (0.84–0.98)	0.91 (0.74–0.97)
Nasal dorsum angle	0.85 (0.72–0.95)	0.85 (0.71–0.90)
Columellar angle	0.89 (0.70–0.98)	0.84 (0.72–0.91)
Columellar–labial angle	0.90 (0.93–0.96)	0.92 (0.90–0.96)
Nasal surface area	0.90 (0.71–0.96)	0.91 (0.85–0.98)
Nasal volume	0.93 (0.79–0.97)	0.92 (0.82–0.95)

ICC, intraclass correlation coefficient; CI, confidence interval.

Precision (the magnitude of the difference between repeated measurements utilizing the same morphometric method by the same rater) was analyzed with two error magnitude statistics^60–65^: the mean absolute difference (MAD) and corresponding relative error magnitude (REM). The MAD across each data set was calculated as the average of absolute differences between the values of two measurement sets. The MAD values were interpreted as follows: highly precise if <1 mm/degree, precise if 1–1.9 mm/degree, or less precise if >2 mm/degree. The REM was calculated by dividing the MAD for a given parameter by the grand mean for that parameter, multiplied by 100. The REM scores were divided into 5 agreement categories: error magnitude <1% = excellent, 1% to 3.9% = very good, 4% to 6.9% = good, 7% to 9.9% = moderate, and >10% = poor.

The MAD values and REM scores were included in Table 8. The MAD values were deemed highly precise for all 4 linear, 4 angular, 1 surface area, and 1 volume parameters, with no MAD value determined as precise or less precise. REM scores were deemed excellent or very good (90% and 10% of parameters, respectively) for all 4 linear, 4 angular, 1 surface area, and 1 volume parameters, with no REM score determined as moderate or poor.

**Table 8:** Error statistics for 3D Nasal Morphometric Measurements Averaged Across all Linear, Angular, Area, and Volume Parameters.

Parameters	Intrarater		Interrater	
	MAD*	REM**	MAD*	REM†
**Linear measurements**				
Mean	0.33 mm	0.55	0.41 mm	0.68
Minimum	0.28 mm	0.20	0.30 mm	0.52
Maximum	0.41 mm	0.70	0.47 mm	0.87
**Angular measurements**				
Mean	0.45°	0.61	0.47°	0.73
Minimum	0.37°	0.21	0.42°	0.32
Maximum	0.48°	0.92	0.49°	1.15
**Nasal surface area**	0.34 cm^2^	0.63	0.43 cm^2^	0.81
**Nasal volume**	0.39 cm^3^	0.58	0.40 cm^3^	0.73

mm, millimeters; cm, centimeters; ° degrees; MAD, mean absolute difference; REM, relative error magnitude; All REM values are presented as a percentage of the grand mean; * All MAD values were deemed highly precise for all 4 linear, 4 angular, 1 surface area, and 1 volume parameters; ** All REM scores were deemed excellent for all 4 linear, 4 angular, 1 surface area, and 1 volume parameters; † All REM scores were deemed excellent for all 4 linear, 2 angular, 1 surface area, and 1 volume parameters, with exception of nasal dorsum angle (1.15%) and columellar angle (1.08%) parameters with very good scores.

### Statistical analysis

Mean, standard deviations, and 95% confidence intervals were used for metric variables and percentages were used for categorical variables to carry out descriptive analysis. The Kolmogorov–Smirnov test revealed that the data were not normally distributed, and the non-parametric tests, Wilcoxon signed-rank and Kruskal–Wallis, were performed to assess the comparisons. Spearman’s correlation test was used for the correlation analyses^66^. A Bonferroni correction was applied for multiple comparisons. Correlation coefficients were interpreted as follows: high if *r* > 0.70, moderate if *r* = 0.30 to 0.70, or low if *r* < 0.30. Linear regression analysis was performed to test whether the age, gender (male versus female), and group (cleft cohort versus dentofacial cohort) presented any relationship with the FACE-Q and nasal morphometric parameters. Two-sided values of *p* < 0.05 were considered statistically significant. All analyses were performed using SPSS Version 23.0 (Chicago, IL, USA).

### Meeting presentation

Awarded for best cleft-related research presentation at the 9^th^ Asian Pacific Cleft Lip-Palate & Craniofacial Congress and the 12^th^ Annual Meeting of THAICLEFT, in Khon Kaen, Thailand; November 11–12, 2019.

## Supplementary information

Supplementary Fig. S1 to S4.
