# Supplementary material for: Patient- and 3D morphometry-based nose outcomes after skeletofacial reconstruction
Source: Sci Rep. 2020 Mar 6;10:4246. doi: 10.1038/s41598-020-61233-6 (PMC7060327; doi:10.1038/s41598-020-61233-6)
Supplement: Supplementary file 1 — Supplementary Fig. S1 to S4. [file 41598_2020_61233_MOESM1_ESM.pdf]

Patient- and 3D morphometry-based nose outcomes after skeletofacial reconstruction

Rafael Denadai, Pang-Yun Chou, Hyung Joon Seo, Daniel Lonic, Hsiu-Hsia Lin, Betty CJ Pai & Lun-Jou Lo

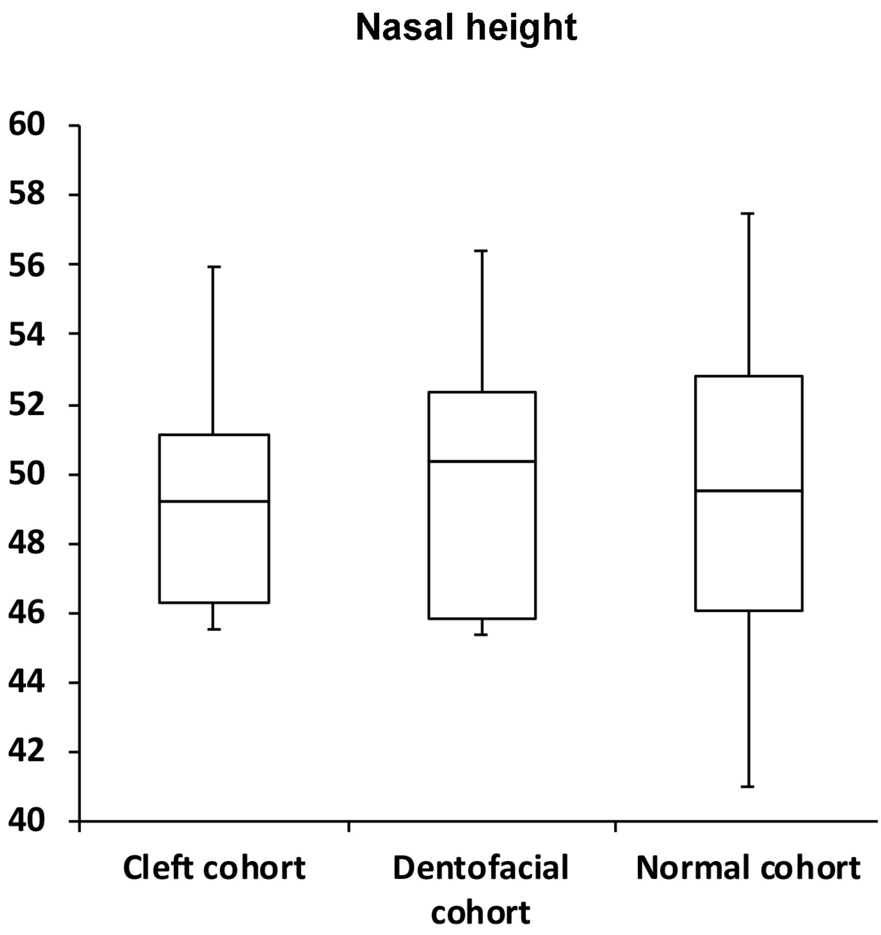

Supplementary Fig. S1. Box plots demonstrating the distribution of 3D nasal height values in the cleft, dentofacial, and normal cohorts.

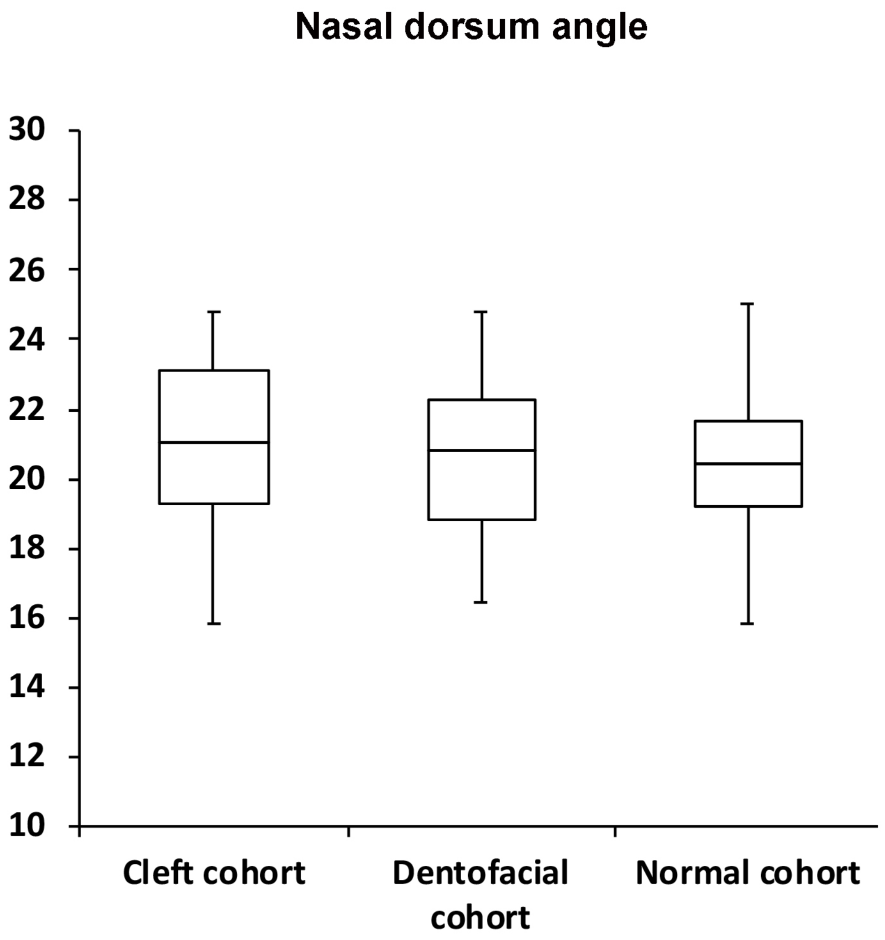

Supplementary Fig. S2. Box plots demonstrating the distribution of 3D nasal dorsum angle values in the cleft, dentofacial, and normal cohorts.

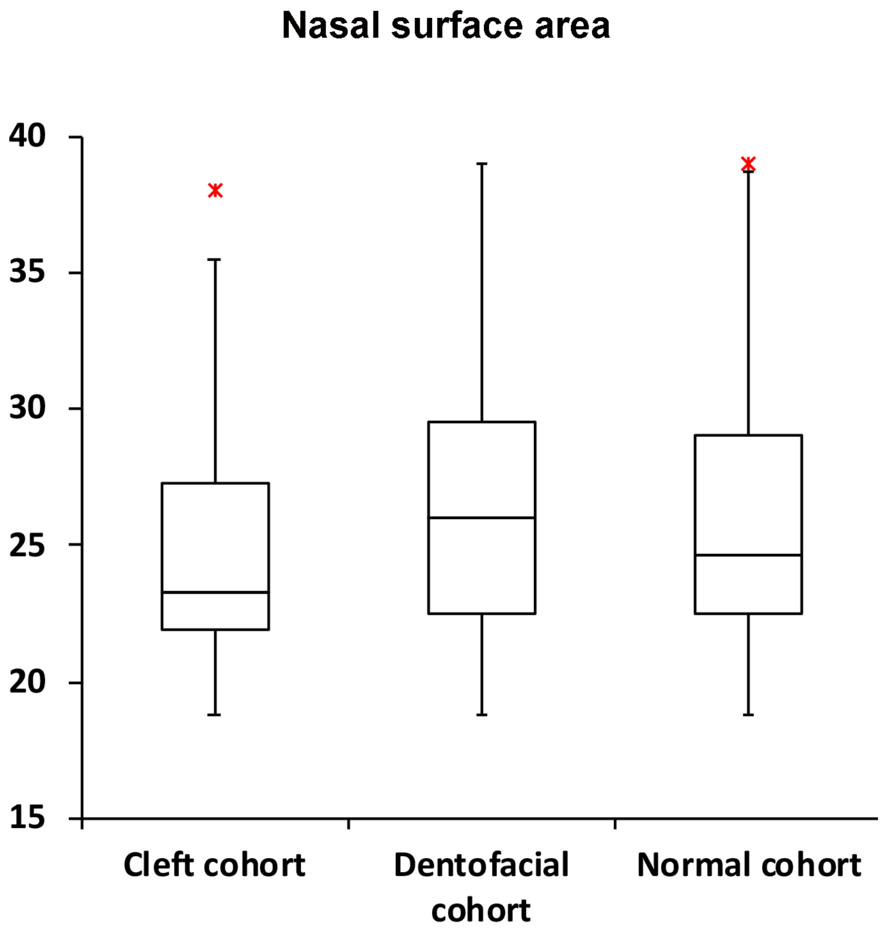

Supplementary Fig. S3. Box plots demonstrating the distribution of 3D nasal surface area values in the cleft, dentofacial, and normal cohorts. Red asterisks indicate maximum outliers' values.

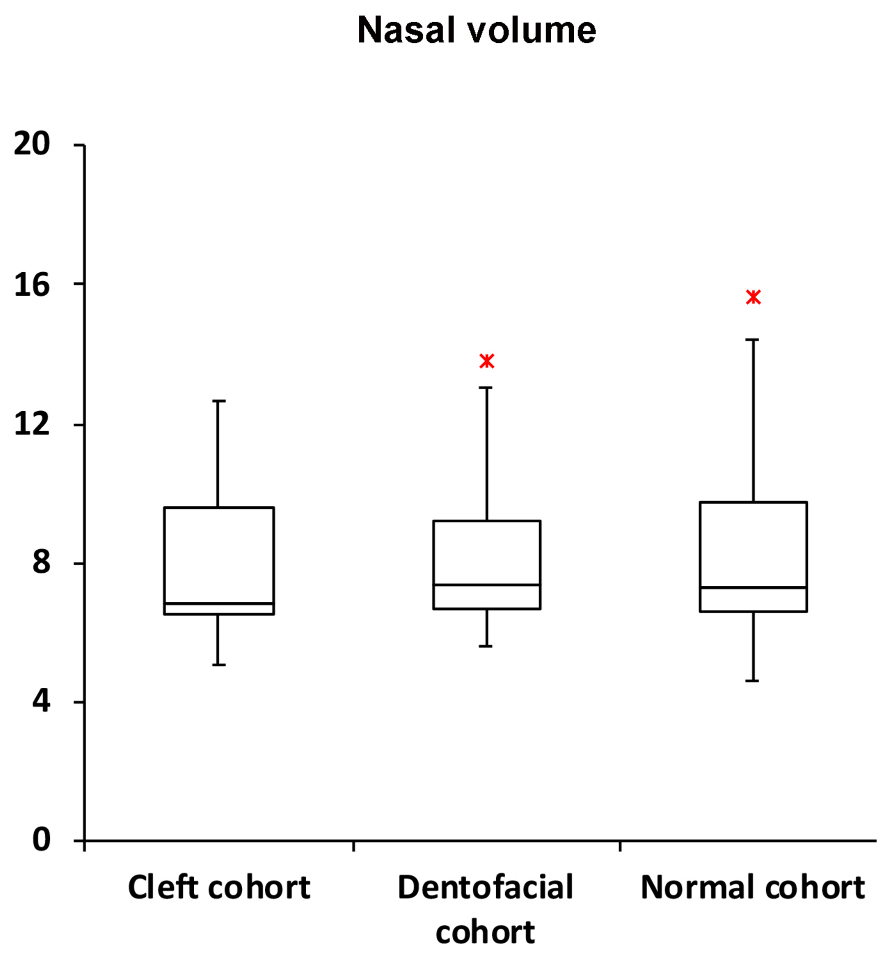

Supplementary Fig. S4. Box plots demonstrating the distribution of 3D nasal volume values in the cleft, dentofacial, and normal cohorts. Red asterisks indicate maximum outliers' values.
